# Integration of Remote Sensing and Mexican Water Quality Monitoring System Using an Extreme Learning Machine

**DOI:** 10.3390/s21124118

**Published:** 2021-06-15

**Authors:** Leonardo F. Arias-Rodriguez, Zheng Duan, José de Jesús Díaz-Torres, Mónica Basilio Hazas, Jingshui Huang, Bapitha Udhaya Kumar, Ye Tuo, Markus Disse

**Affiliations:** 1 Chair of Hydrology and River Basin Management, Technical University of Munich, 80333 Munich, Germany; monica.basilio@tum.de (M.B.H.); jingshui.huang@tum.de (J.H.); bapitha.udhayakumar@mytum.de (B.U.K.); ye.tuo@tum.de (Y.T.); markus.disse@tum.de (M.D.); 2 Department of Physical Geography and Ecosystem Science, Lund University, S-223 62 Lund, Sweden; zheng.duan@nateko.lu.se; 3 Center for Research and Assistance in Technology and Design of the State of Jalisco, Colinas de la Normal, 44270 Guadalajara, Jalisco, Mexico; jdiaz@ciatej.mx

**Keywords:** Landsat 8 OLI, Sentinel 2 MSI, Sentinel 3 OLCI, water quality monitoring system, extreme learning machine, support vector regression, inland waters, turbidity, Chlorophyll-a, secchi disk depth

## Abstract

Remote Sensing, as a driver for water management decisions, needs further integration with monitoring water quality programs, especially in developing countries. Moreover, usage of remote sensing approaches has not been broadly applied in monitoring routines. Therefore, it is necessary to assess the efficacy of available sensors to complement the often limited field measurements from such programs and build models that support monitoring tasks. Here, we integrate field measurements (2013–2019) from the Mexican national water quality monitoring system (RNMCA) with data from Landsat-8 OLI, Sentinel-3 OLCI, and Sentinel-2 MSI to train an extreme learning machine (ELM), a support vector regression (SVR) and a linear regression (LR) for estimating Chlorophyll-a (Chl-a), Turbidity, Total Suspended Matter (TSM) and Secchi Disk Depth (SDD). Additionally, OLCI Level-2 Products for Chl-a and TSM are compared against the RNMCA data. We observed that OLCI Level-2 Products are poorly correlated with the RNMCA data and it is not feasible to rely only on them to support monitoring operations. However, OLCI atmospherically corrected data is useful to develop accurate models using an ELM, particularly for Turbidity (R^2^ = 0.7). We conclude that remote sensing is useful to support monitoring systems tasks, and its progressive integration will improve the quality of water quality monitoring programs.

## 1. Introduction

Inland waters, as a source of good water quality, are essential to human health. The amount of worldwide population relying on surface water for drinking purposes ranges between 70 and 85% [1]. Additionally, surface waters provide services such as irrigation, fisheries for food, hydropower, purification of wastewaters, flood protection, wetland plants for fuel and construction, as well as water and nutrient cycling provided by surface waters [2]. The impact of human anthropogenic activities such as discharge of waste products or increased loads of nutrients and sediments from agriculture and urban areas escalate the eutrophication of global inland waters. This situation raises concerns about the protective measures of inland water resources and how to ensure their adequate environmental quality. A fundamental task to understand and prevent environmental threats is the continuous monitoring of water quality. The information gathered during monitoring is used to warn of current and emerging risks and assent of applicable regulations by pointing to changes in trends of quality parameters. From monitoring, empirical data is provided to aid decision-making on health issues, and it provides evidence for water quality management in the long term. Currently, monitoring water quality is a growing challenge because of the difficulty in costs and time resources of sampling tasks and identifying a large number of chemicals for industry and domestic uses that make their way into inland waters. Nowadays, every country is responsible for the state of its water. In developing countries, the priority has been to supply drinking water and control wastewater. In these cases, water quality monitoring programs are designed to be conducted with conventional, boat-based, or buoy-based measuring techniques at specific times and locations and their subsequent laboratory analysis. Some national monitoring programs for inland waters are already under continuous development and operation. 

In Latin America, Mexico has established a national water monitoring network (RNMCA) since 1996. Initially, with 200 stations and a sampling frequency of 2 to 3 campaigns a year for lakes, it has gradually been expanded to operate with more regularity after a major renovation in 2012. Today, 2700 stations integrate a surface water dataset with information about the location of the stations and measurement frequency. In Brazil, a similar number of stations (4500) were planned to be reached by 2020 [3], but other cases are still in need of improvement, such as Argentina with 617 stations [4] or Chile, where until 2009, it lacked a coordinated monitoring system at a national level [5]. However, even with the improvement in such cases, the coverage in spatial and temporal scales of the water monitoring programs is limited by the economic costs of each sampling station and the frequency of measurement. Remote sensing offers a strong potential to monitor water quality in inland waters because it magnifies forthcoming data availability by providing radiometric measures prone to be associated with water quality parameters. Mainly visible (VIS) and near-infrared (NIR) bands of the electromagnetic spectrum have been used in several studies to obtain correlations between radiometric data acquired from sensors on board satellites and physical and biochemical constituents in water [6,7,8,9,10,11,12,13]. As a result of many years of research, the UN Environment Project recognizes the need to integrate remote sensing sensors in the water quality monitoring tasks [2].

To reliably establish such relation from modeling, radiometric values and in-situ water quality measurement should be acquired in a coincident acquisition date. Models capable of finding a relationship between radiometric data from sensors and water quality constituents can be classified as empirical, semi-analytical, or machine learning-based [14]. Empirical models fit a standard linear regression between spectral radiometric values in the form of bands or band ratios from the sensor and in-situ water quality measurements. These models are simple and transparent in their process, requiring minimal computational requirements. However, they are limited to the range and temporal scale of the input data because weather conditions and water conditions create significant alterations in observed radiometric data, bounding its regional generalization. Semi-analytical models are based on the optical properties of the water and the atmosphere, which are unrelated to the light field and are therefore called inherent optical properties (IOPs). These IOPs are used to calculate absorption and backscattering coefficients from which water quality parameters can be retrieved. Because of its physics background in the properties of water and atmosphere, these models are generalizable on a regional scale. However, there is a need for extensive in-situ data for validation. The required information about atmospheric composition and bottom reflectance makes its application difficult where this data is missing [15]. Machine learning (ML) incorporates the advantages of empirical modeling but with an increased computational capacity to handle complex nonlinear relationships. Similar to empirical methods, ML algorithms are limited by the range and settings of input data of its trained models. However, they present several advantages such as iterative learning to reduce the overall error and to maximize fit [16]. Due to its novelty, the use of ML is still not well understood in water quality retrievals, and its application is still necessary to further understand its behavior in remote sensing of inland waters [17]. 

Several sensors are available for potential applications in water quality retrievals to supply these varieties of models with input data. The Operational Land Imager (OLI) onboard NASA’s satellite Landsat-8 (launched 2013) has a broad background of applications in inland waters through the former Landsat missions [11,18,19,20,21,22,23]. Despite its original design for terrestrial applications, it is suited to inland waters due to its spatial and spectral resolution (11 spectral bands, up to 30 m spatial resolution) and with the drawback of a sparse temporal resolution for regular monitoring (16 days) [24]. The use of Medium Resolution Imaging Spectrometer (MERIS) (15 bands, 300 m resolution) on board the European Space Agency (ESA) ENVISAT contributed to monitoring inland waters from 2002 to 2012 [8,17,25,26] and its archives still offer a potential data mine for further applications. The ESA designed the Ocean and Land Color Instrument (OLCI) on board the Sentinel-3 with similar and improved characteristics (21 spectral bands, up to 300 m spatial resolution) is expected to assume the legacy of MERIS and continue with suitable applications on monitoring inland waters. The MultiSpectral Instrument (MSI) onboard Sentinel-2 has suitable characteristics for water quality monitoring (13 spectral bands, up to 10 m spatial resolution) and temporal resolution (10-days single and 5-days combined constellation revisit frequency of Sentinel-2A and Sentinel-2B). Chlorophyll-a (Chl-a) concentrations have been recently investigated with MSI in different locations worldwide such as Estonia [27] or Africa [28]. The utilization of geographic information systems (GIS) is a key resource to gather and manage field and remote sensing data. GIS merges different types of data into a common framework where layers of information are displayed to detect patterns and relations. These observations are useful to communicate, analyze and take decisions to solve complex problems. For monitoring, GIS plays a key role, because of the clear manner the changes can be detected using a variety of data [29]. When monitoring inland waters by remote sensing, the patterns of water parameters are retrieved from models using sensors’ data and they are commonly displayed in spatial and temporal scales, represented in maps of spatial distribution [30]. 

Despite the available approaches in computational modeling and remote sensing data, the consideration of such techniques when planning and executing tasks in water quality monitoring is limited. Consequently, remote sensing may not be recognized as the main driver of the design of water quality monitoring programs and decisions of water managers. This may be because local managers are not considering technical expertise in remote sensing techniques and because research integrating data from entire water monitoring programs for modeling purposes is scarce [31]. Therefore, an evaluation of remote sensing techniques using data from water quality monitoring programs is necessary as an initial step to foster the integration of remote sensing data into the monitoring routines. This work addresses this situation using the RNMCA in Mexico as a case study, acquiring entire time series of relevant-remote-sensing water quality parameters. This data is matched with available remote sensors and modeled through machine learning approaches to evaluate the feasibility of integrating existing monitoring data into predictive models. Additionally, we provide suggestions to improve monitoring programs with the progressive integration of remote sensing. 

The specific objectives of this study are: (1) to verify the feasibility to use existing data (gathered with no considerations of remote sensing) from monitoring programs in a routine of water quality parameter retrievals by remote sensing; (2) evaluate readily-to-use (Level 2 Products) water quality remote sensing products with respect to historical water quality measurements; (3) use radiometric data from available sensors and machine learning techniques for water quality parameters estimations; (4) find feasible water quality parameters and inland waterbodies for such monitoring routine. Additionally, it is provided a critical opinion of the main limitations and challenges when integrating these two independent sources of data. This work highlights the need of upscaling this research field using national-wide monitoring data, evaluating different available sensors, and applying multitemporal analysis with the availability of the sensor’s archives. 

## 2. Study Areas

**Figure 1:**
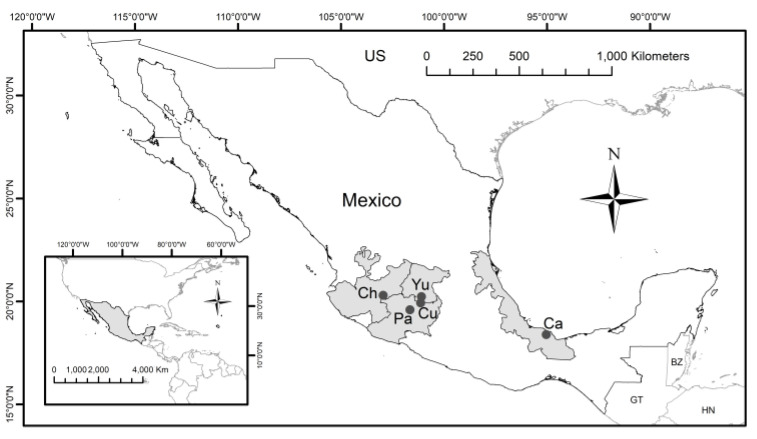
Regions and locations of the studied lakes in Mexico. Ch: Chapala, Cu: Cuitzeo, Pa: Pátzcuaro, Yu: Yuriria, Ca: Catemaco.

**Figure 2:**
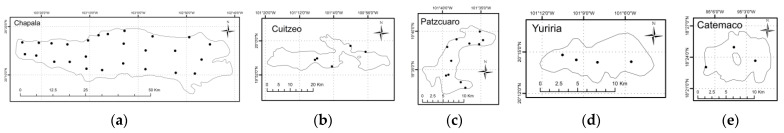
Location of the RNMCA sampling stations (black dots) in the lakes: (**a**) Lake Chapala, (**b**) Lake Cuitzeo, (**c**) Lake Pátzcuaro, (**d**) Lake Yuriria, and (**e**) Lake Catemaco. The scale is different for each lake.

We study five Mexican lakes identified by the Mexican water authority as the most relevant ones in terms of size and regional use, therefore we considered them as priority targets in terms of the integration of monitoring systems with remote sensing: Chapala, Cuitzeo, Pátzcuaro, Yuriria, and Catemaco [32]. These are all located in the Trans-Mexican Volcanic Belt (TMVB) and have a volcanic origin, with the exception of the lake of Yuriria, which is artificial. Catemaco belongs to the Gulf-Center hydrological-administrative region, and the other four lakes are within the Lerma-Santiago-Pacific area (Figure 1). The sampling stations of the RNMCA are displayed in Figure 2. 

Chapala Lake is the largest inland lake in Mexico. It covers approximately 3% of its territory with an area of 1116 km^2^, and it is considered one of the largest and shallowest tropical lakes in the world [32]. It is located at 1523.8 m.a.s.l. at 19°05′–21°03′ N and 99°22′–103°31′ W. It has a mean depth between 4 and 6 m with a maximum depth of 8 m. Its dimensions are 75 km in length and 5.5–20 km in width [33,34]. The lake’s primary input is precipitation, but it also receives water from the water sheet and several streams, the Lerma River being its main tributary. Evaporation, pumping, and the Santiago River are the main outflows [35]. The lake’s catchment area is a mixture of lacustrine sediments with volcanic rocks and basaltic and andesitic lavas accumulated since the Miocene. Thermal springs, outcrops, and calcareous sinter are also present in the basin [36]. The weather in the catchment is mainly humid subtropical, with a mean annual precipitation of 730 mm and a uniform temperature around 24 °C [34]. Chapala lake has a high level of sediments and turbidity, partly by the geology and topology of the area that facilitates the transport of clay particles to the lake. In particular, the Lerma River can carry many sediments from areas affected by erosion [33,36]. Due to intense water extraction, dry periods, and land-use change, the lake’s volume has decreased up to 42% [35]. In addition, the rivers and streams can transport contaminants from industrial, agricultural, and livestock activities in the catchment area [33,34]. 

Located at 1820 m.a.s.l with coordinates 20°05′–19°52′ N and 100°50′–101°19′ W, Lake Cuitzeo is the second largest lake in the country by surface area [37,38]. With a maximum potential area of 420 km^2^, currently, Cuitzeo Lake consists of brackish waters of 1–2 m of depth over an area fluctuating around 300 km^2^ [39,40]. The lake is highly susceptible to weather variations and has been closed to desiccation during at least three severe drought periods in the last century [38]. The approximately 4000 km^2^ watershed has several low and high hills originated by volcanic activity during the Miocene and Pliocene, including pyroclastic-fall deposits and fluviolacustrine plains [39]. The Grande and Queréndaro Rivers are the main tributaries [37]. There is no natural outlet in the lake, although according to Soto-Galera [41], it could have been connected to the Lerma River during the Holocene. The climate in the catchment is moderate, with temperatures ranging from 10 to 28 °C. Annual precipitation can vary from 765 to 1200 mm and it is concentrated in the summer, from May to October [37,39,41]. As the quality and quantity of the water feeding the lake have decreased (e.g., waters coming from municipal and industrial activities or agricultural runoffs), the lake is in a hypertrophic state. Furthermore, it also has detectable arsenic levels coming from geothermal boreholes around the lake and a thermal spring located on a magmatic chamber [37]. 

Pátzcuaro Lake is located at 19°32′–19°42′ N and 101°32′–101°42′ W and 3035 m.a.s.l. It has a maximum surface area of 116 km^2^ with an average depth of 5 m, although certain zones can have up to 12 m [42]. The lake and its four islands originated from volcanic activity during the Pleistocene about 1 million years ago [43]. The lake is well mixed, not stratified, and it is maintained mainly by small springs of shallow groundwater and by local runoff [44,45]. The drainage basin covers 929 km^2^ and, while the system today is endorheic, it could have drained to the Lerma River 25,000 years ago. Two seasons dominate the weather: rainfall in summer and stable dry conditions in winter with a mean annual precipitation of 950 mm [45]. Pátzcuaro Lake has been subject to several paleoenvironmental studies where the extracted cores contain lacustrine sediments that record climate change, human impact, volcanic activity and earthquakes for periods up to 48,000 years ago [43,44,45,46]. In recent years, fish biodiversity in the lake has decreased due to anthropogenic activities [42,44,45]. 

Yuriria Lake is located at 20°13′–20°17′ N and 101°12′–101°03′ W at 1740 m.a.s.l. [47]. With 13.79 km in length and 5.88 km wide, it has a surface of 66 km^2^ and a maximum depth of 3.2 m [48]. It is an artificial lake considered the first post-Columbian hydraulic work, as it was formed after building a deviating water channel from the Lerma River in 1548. The silty clay on the surface avoids water leakage to the aquifer [47]. The channel from the Lerma river is still the main tributary of the lake [48], although precipitation and runoff also contribute to it. The mean annual temperature in the area is 18 °C and the rainy season is from May to September, with annual precipitation that can vary from 669 to 797 mm. The lake supports migratory and resident birds, and the area is considered a Wetland of International Importance (RAMSAR) since 2004 [47]. Espinal Carreón et al. [48] identified eutrophication and contamination levels that may be dangerous for fish biodiversity and recreation. 

Catemaco lake is located at 322 m.a.s.l. with coordinates 18°21′–18°27′ N, and 95°01′–95°07′ W, between San Martín Tuxtla Volcano and the Sierra de Santa Marta. It is part of the subcatchment of the San Juan River, a tributary of the Papaloapan River, the second most fast-flowing river in Mexico [49]. With an approximately squared layout, Catemaco Lake has an area of about 75 km^2^. The mean depth is 7.6 m, but while the lake basin is mainly a plateau of 11 m deep maximum, there are three pits that reach up to 22 m depth [50]. The lake receives water from at least 10 tributaries, and it is also fed by groundwater and precipitation, which can be up to 5000 m per year. Its main effluent is the Grande de Catemaco River, a tributary of the San Juan River [51,52]. Catemaco Lake is considered a warm polymictic lake, there is no stratification, and the concentration of dissolved oxygen is constant across the water column. The light penetration between 0.53 and 2 m depth and its temperature ranges from 23 to 28 °C [53]. The catchment area of Catemaco covers 322.2 km^2^. It has escarpments, cinder cones, and maars resulting from volcanic activity in the late Miocene (~7 million years ago) and having the latest eruptions in the XVIII century. In fact, the lake formed when several cinder cones blocked the drainage to the north, and the lake contains many islands formed by subaquatic vulcanism [54]. Catemaco Lake is in the tropical rain forest and has high biodiversity. Divided by the NW–SE axis, approximately half of the lake borders with the Natural Reserve of Los Tuxtlas [51]. However, the area is affected by deforestation, water abstraction, and water pollution due to agriculture and livestock farming [51]. With coliform, organic matter, hydrogen sulfur, water lilies, and phosphorous, the lake has been classified as eutrophic [52].

In general, the lakes are affected by well-known stressors caused by anthropogenic activities. Furthermore, they are exposed to a certain degree of diversions and removals of water for agricultural, livestock, and industrial activities [33], numerous discharges of untreated industrial and municipal wastes, and a growing urban population [41]. This has disruptive effects, such as drying up and refilling by sediments from erosion and runoff from deforested uplands due to poor management of soil resources [48], loss of surface area, reduction of the water column, lower water transparency and hyper-eutrophication, erosion, or nutrient loads [53,55]. As the lakes are surrounded by large urban areas or are close to industrially developed regions, the spectral signature is contaminated to some degree by atmospheric effects caused by aerosols and other gases. Hence, the optical properties and identification of various optical water types are challenging. 

## 3. Materials and Methods

### 3.1. In-Situ Data

This work utilizes the dataset available from the national water monitoring network (RNMCA) managed by the Mexico’s national water council (CONAGUA), which is the primary source of water quality data in the country. The historical-series data contain daily physic-chemical data taken in different intervals between 2012 and 2018 using in-situ field campaigns with exact sampling dates. The information is open access under http://sina.conagua.gob.mx/sina/ (accessed on 8 June 2020) The water quality measures are taken on monitoring stations operated by CONAGUA all over the country, which in 2019 had more than 4000 fixed locations. The biggest lakes of the country analyzed in this study counted with 30 in Chapala, 15 in Cuitzeo, 10 in Pátzcuaro, 8 in Yuriria, and 4 in Catemaco, and frequency of measurement is also limited to 2 to 3 times per year. From the available parameters, this study focuses on Chlorophyll-a (Chl-a), Turbidity, Total suspended matter (TSM), and Secchi disk depth (SDD) as these are important for water quality and present in remote sensing of inland waters studies [14,24,56]. 

CONAGUA manages the RNMCA to obtain the water quality parameter following national and international standards to the parameter determinations. In this sense, the Chl-a measurement derived from the extraction method 10200-H described in the American Public Health Association [57]; turbidity determination follows the nephelometry method referred in the NMX-AA-038-SCFI-2001 [58], while the TSS are determinate under the Mexican standard NMX-AA-034-SCFI-2015 [59] procedures; SDD is measured following the 30 cm Secchi Disk procedure [57]. These Mexican standards follow the general criteria for controlling the quality of analytical results from NMX-AA-115-SCFI-2015 [60]. When the usage of water is for the supply of drinking water, the relevant norm for sampling in surface and groundwater for water quality parameters is the NOM-014-SSA1-1993 [61] which indicated measures should be taken with the bottle immersed in the water with the neck facing down, up to 15 to 30 cm deep. Unfortunately, no measurements of radiometric data are available from RNMCA. Descriptive statistics of the water quality dataset are shown in Table A1.

### 3.2. Satellite Data and Processing

The general characteristics of the sensors used in this study are shown in Table 1. Landsat-8 OLI multispectral images were downloaded from the United States Geological Service (USGS) website (earthexplorer.usgs.gov (accessed on 10 June 2021), Collection 1 Level 2, on-demand products). Sentinel-3 OLCI Level 1 Full Resolution images were downloaded from the European Organization for the Exploitation of Meteorological Satellites EUMETSAT Data Centre website https://archive.eumetsat.int/usc/ (accessed on 10 September 2020). Sentinel-2 MSI Level-1C (L1C) images were downloaded from the Copernicus Open Access Hub https://scihub.copernicus.eu/dhus/#/home (accessed on 12 October 2020) using the Sen2r package [62]. Additionally, Sentinel-3 OLCI Level-2 Full Resolution Water Products (OLCI WFR) were considered for further comparison of its Chl-a and TSM layers. Table A2 displays the selected bands from the sensors used in this study. 

Synchronized field and satellite data were identified with the allowance of ±3 days of difference. For OLI, 41 matches were found, while 47 for OLCI and 31 for MSI. The number of sampling points present on each image and more details of the synchronized data are shown in Table A3. Pixel averaging was not considered since the sampling stations are well located and the resolution is considered adequate for all the sensors [33].

OLI Collection 1 Surface Reflectance includes the use of the Land Surface Reflectance Code (LaSRC) (version 1.4.1), which produces Top of Atmosphere (TOA) Reflectance and TOA Brightness Temperature (BT) using calibration parameters from the metadata. These TOA products are further corrected with water vapor and ozone data from the Moderate Resolution Imaging Spectroradiometer (MODIS) and digital elevation derived from the Earth Topography Five Minute Grid (ETOP05) to generate surface reflectance (SR) [22]. Atmospheric correction of OLCI L1b radiances was based on the well-known Case 2 Regional CoastColour (C2RCC) processor available as plug-in in SNAP (v7.0). This selection was made based on the standard and recurrent application of the C2RCC in literature and its use as a standard atmospheric corrector, which helped develop the methodology with certainty. The C2RCC retrieves directly remote sensing reflectance (Rrs). Sentinel-2 MSI images were resampled to 60 m with the Resampling (v2.0) tool to give the same base for each AC processor. Similarly, C2RCC (v0.15) was applied to Sentinel-2 products and the retrieved Rrs. 

### 3.3. Modeling Methodology

Dimensionality reduction was necessary due to the many scenarios to analyze with different lakes (5), sensors (3), algorithms (3), hyperparameters and bands as predictors, and the cross-validation necessary on each model. Selecting a subset with the relevant predictors was desirable to prevent overfitting and enhance the generalization and avoid collinearity conditions, commonly present in nearby bands situated next to each other [63]. The advantages of this reduction are fewer training periods and fewer computational demands. Investigated bands were inside the VIS, and NIR regions as these are well documented for having spectral relation to the studied water parameters [14,64] and work well when developing algorithms for Case 2 waters. To gain hints about further relevant wavelengths to use for every sensor, the correlation between bands and field data was inspected utilizing its distribution and scatterplots. Attributes were also tested with logarithmic, exponential, and cubic transformation for visual analysis. Linear relationships between predictors and parameters were using Pearson’s correlation. The correlations were further displayed in a heatmap matrix to inspect further and enhance the most correlated bands. The bands of every sensor with stronger correlation to the field data were selected to further be analyzed as input for the models. 

Different ML algorithms were trained with the field and radiometric data. Recent trends are focused on applying neural network techniques in the remote sensing of inland waters [23,65,66] for its robust results and capacity to detect nonlinear patterns between radiometric data and water quality parameters. A novel approach is the extreme learning machine (ELM). Its basic form is a feedforward neural network with a single hidden layer that adjusts randomly the weights of the hidden layer with no iterative optimization, reducing the computational demand of a traditional feedforward neural network [67]. With a *d* number of nodes, *L* as the input layer and *m* as the output layer, for training samples as ${\left\{\left(x_{i},t_{i}\right)\right\}}_{i=1}^{N}$, where $x_{i}=\left[x_{i1},x_{i2},\cdots ,x_{id}\right]\;\in R^{d}$, the matrix of the network is expressed as:

(1) Hβ=T

being

(2) $$H={\left[\begin{array}{ccc} g\left(w_{1},b_{1},x_{1}\right) & \ldots & g\left(w_{L},b_{L},x_{1}\right)\\ \vdots & \vdots & \vdots \\ g\left(w_{1},b_{1},x_{N}\right) & \vdots & g\left(w_{L},b_{L},x_{N}\right) \end{array}\right]}_{NxL},\;\beta ={\left[\begin{array}{c} \beta_{1}^{T}\\ \vdots \\ \beta_{L}^{T} \end{array}\right]}_{Lxm}\;\mathrm{and}\;T={\left[\begin{array}{c} t_{1}^{T}\\ \vdots \\ \beta t_{L}^{T} \end{array}\right]}_{Nxm}$$

where $w_{i}={\left[w_{i1},w_{i2},\cdots ,w_{id}\right]}^{T}$ is the vector of the input weights of the node i and $b_{i}$ is the bias, with both $w_{i}$ and $b_{i}$ randomly generated; $\beta_{i}={\left[\beta_{i1},\beta_{i2},\cdots ,\beta_{im}\right]}^{T}$ is the output weight vector and *g* the activation function. The least-square solution of the outputs weights in the hidden layer is found to train the ELM. The *β* is expressed as:

(3) $$\beta =H^{†}T,$$

where $H^{†}$ is the Moore–Penrose generalized inverse of *H* [67]. 

Hyperparameters to tune for the ELM are the number of neurons in the hidden layer and the activation function. We evaluated different logarithmic ranges for the number of hidden neurons. All the available activation functions [68] for the ELM implementation were evaluated for the different options of hidden neurons. 

Additional algorithms were tested to gain insights into the ELM performance against algorithms with previous applications in the region. Support vector regression (SVR) and least-squares linear regression (LR) demonstrated a good performance when retrieving Turbidity and SDD [64,69,70,71,72,73] in central Mexico [17]. Both SVR and LR algorithms have been applied in predicting water quality parameters with successful results, and their use starts to be common in the evaluation of ML approaches. To tune the hyperparameters of the SVR, the radial basis, sigmoidal and linear kernel together with the regularization parameter and the kernel coefficient in logarithmic ranges were evaluated. The rest of the hyperparameters were used as default. More details of the SVR can be found at Vapnik et al. [74] or in previous applications [17].

To search the optimal hyperparameters of each algorithm, a grid of predefined values was analyzed using leave-one-out cross-validation (LOOCV) to evaluate all possible combinations due to the limited matches between field and satellite data used in training and validations. In this process, a score of performance is calculated for each set, and a later function displays the values reaching the highest score to select the most adequate hyperparameters combination. The individual and synergistic behavior of the investigated bands was evaluated, analyzing all the possible combinations of the remaining predictors by implementing a power set (PS) to perform a spectral sensitivity analysis to finally select the number and kind of predictors retrieving the best error metrics. The power set is defined as follows:

(4) $$PS\left(b\right)=2^{b},$$

where *b* is the number of predictors for that specific dataset. 

After dimensionality reduction, tuning of hyperparameters and optimal number of predictors, the ELM, SVR and LR models were evaluated against each other using their best configurations through a LOOCV for each sensor, lake, and parameter. The training size used for each LOOCV varied depending on the resulting dataset of each lake and sensor, and a correlation analysis, and for all cases it was equal to $n_{samples}-1$. From the three models, the one with best performance was selected as the ideal to model the specific parameter used for a specific sensor. For this we use different controlling metrics, the coefficient of determination (R^2^), the root mean squared error (RMSE), and the mean absolute error (MAE), defined as:

(5) $$RMSE\;\left(y,\;\hat{y}\right)=\sqrt{\frac{1}{n_{samples}}\sum_{i=0}^{n_{samples^{-1}}}{\left(y_{i}-{\hat{y}}_{i}\right)}^{2}},$$

(6) $$R^{2}\left(y,\;\hat{y}\right)=1-\frac{\sum_{i=0}^{n_{samples^{-1}}}{\left(y_{i}-{\hat{y}}_{i}\right)}^{2}}{\sum_{i=0}^{n_{samples^{-1}}}{\left(y_{i}-{\bar{y}}_{i}\right)}^{2}},$$

(7) $$MAE\left(y,\;\hat{y}\right)=\frac{1}{n_{samples}}\;\sum_{i=0}^{n_{samples^{-1}}}{\left|y_{i}-{\hat{y}}_{i}\right|}^{2},$$

where ${\hat{y}}_{i}$ is the estimated value, $y_{i}$ is the observed value and $n_{samples}$ is the number of samples. Usage of ELM was performed via the Caret library [75] in *R*, which applies the elmNNRcpp package from Mouselimis [76] based on the implementation of Gosso [77]. The base codes of ELM can be found at: https://www3.ntu.edu.sg/home/egbhuang/elm_codes.html (accessed on 12 February 2021). SVR and LR were implemented using the Scikit-Learn library (0.20.1) [78] in Python (v.3.8.3).

## 4. Results

### 4.1. OLCI Water Products Compared to RNMCA

**Figure 3:**
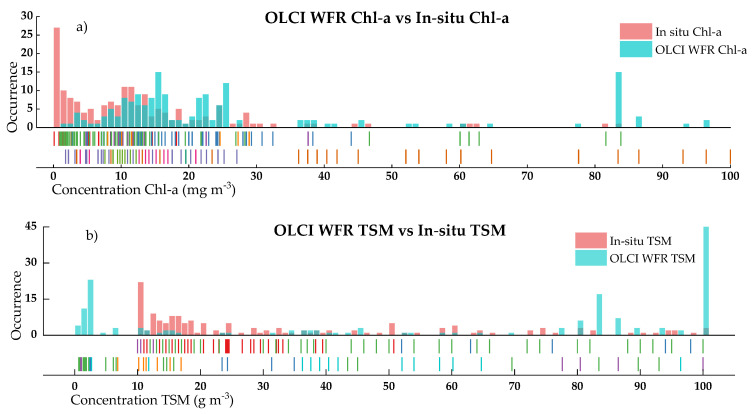
Histograms of OLCI WFR Chl-a (**a**) and TSM (**b**) estimations overlapped to RNMCA measurements. Marks along the x-axis as perpendicular rugs display single qualitative data and its distribution. axes.

Chl-a and TSM values estimated with OLCI WFR were compared against all the in-situ data (Figure 3) to evaluate the possibilities of using these products as part of the monitoring system.

**Figure 4:**
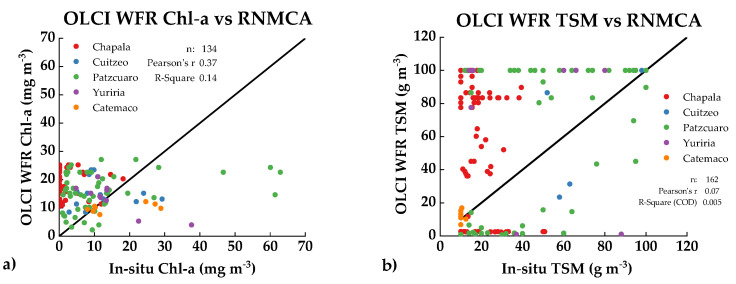
In-situ and S3 derived water quality parameters. (**a**) OLCI WFR Chl-a vs. in-situ measurements, (**b**) OLCI WFR tsm_nn vs. in-situ measurements.

**Figure A1:**
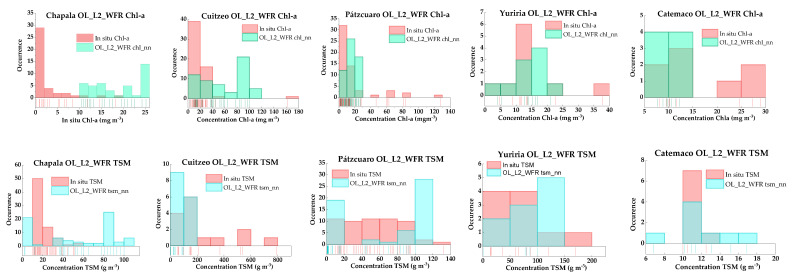
In-situ and S3 derived water quality parameters vs. field data from RNMCA by lake. The upper row shows the Chl-a comparison, the lower row shows TSM.

The overlap between histograms shows significant differences for both water parameters when compared with in-situ data. Chl-a field concentrations are usually low (avg: 14 mg m^−3^) compared to the OLCI products (avg: 30 mg m^−3^). Furthermore, the distribution of the OLCI derivations from Chl-a seems to have a closely normal distribution shape spread in the range 5–30 mg m^−3^. The averages values for field TSM (avg: 53 g m^−3^) and OLCI derived (avg: 53 g m^−3^) are very close to each other; however, the constraints from the TSM layer relies on the frequent prediction of values close to either 0 or 100 g m^−3^, as seen in Figure 2b). The complete overlap of histograms by lake is shown in Figure A1. Figure 4 shows the scatterplots of the OLCI water products in-situ data. The Pearson’s correlation of both data remains low, particularly for TSM (r = 0.07), where the differences of extreme predictors between 0 and 100 g m^−3^ values display scattered values. On the other hand, Chl-a water products were underestimated above 30 mg m^−3^.

Individually, Chl-a retrievals were overestimated in Chapala. Pátzcuaro had little agreement with under and overestimation before and after 15 mg m^−3^. Catemaco showed a better agreement. For TSM, Pátzcuaro, Chapala and Yuriria suffered poor agreement, and Catemaco again showed the best correlations at lower concentrations.

### 4.2. Data Evaluation and Model Performance

**Figure 5:**
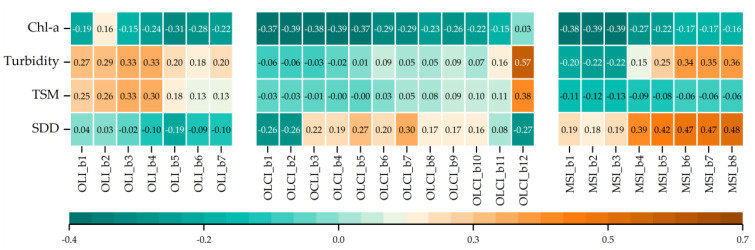
Correlation matrix heatmap of reflectance values of the analyzed bands and water quality parameters. Bands of the Operational Land Imager (OLI) are shown at left, Ocean and Land Color Instrument (OLCI) are shown at the center, and Multi Spectral Instrument (MSI) are shown at the right.

Generally, the exploratory analysis on a single set per sensor containing all the water quality data of each lake did not present strong correlations between reflectance from sensors and RNMCA data (Figure 5). For OLI, higher correlations were seen in the VIS for Turbidity and TSM (R ≈ 0.30) and b5 for Chl-a (R ≈ 0.31). OLCI displayed slightly better correlations in the VIS for Chla (R ≈ 0.38) and b12 for Turbidity and TSM (R ≈ 0.57 and R ≈ 0.38) alongside b1, b2, and b12 for SDD. MSI displayed higher correlations in VIS bands with Chla (R ≈ −0.38), NIR with Turbidity (R ≈ 0.35) and SDD (R ≈ 0.47). TSM showed the weakest correlations in OLCI (R ≈ 0.03) and MSI (R ≈ 0.12) and slightly better for OLCI (R ≈ 0.30). Further analysis of Pearson’s coefficient was performed, analyzing each data set separate by sensor and lake with an additional cleaning process of noise values prior to model training. From a practical point of view, Sentinel-3 OLCI presents the strongest correlation for all the target parameters except SDD.

**Figure 6:**
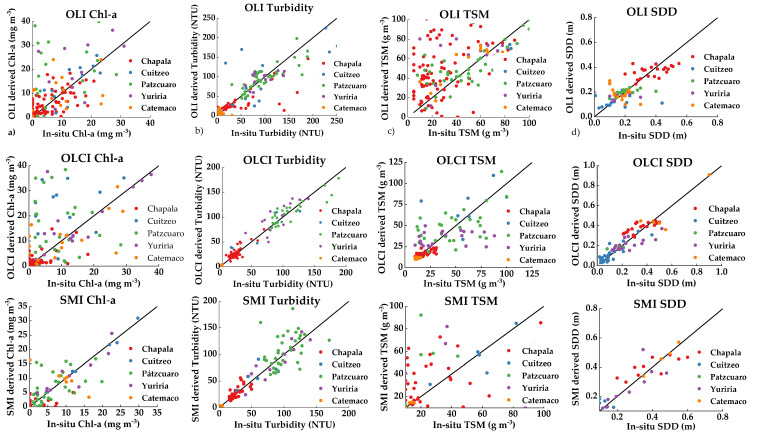
Comparison of estimated Chlorophyll-a (Chl-a, column (**a**)), Turbidity (column (**b**), total suspended matter (TSM, column (**c**)) and Secchi disk depth (SDD, column (**d**)) by sensor. Individual estimations by lake are displayed on each figure: Operational Land Imager (OLI) (top), Ocean and Land Color Instrument (OLCI) (center) and Multi Spectral Instrument (MSI) (bottom).

The average error metrics by lake and parameter are shown in Table 2 and Table 3 as an overview of the performance in error metrics of lakes and water parameters. The complete validation of models displaying error metrics, the best algorithm for each lake, sensor, and water parameter together with tuned hyperparameters and training size is shown in Table A4, Table A5 and Table A6. Scatter plots of all the in-situ parameters and best models for each sensor resulting from the LOOCV are shown in Figure 6. Modelled algorithms varied in every sensor and lake depending on the approach and hyperparameters. The additional factor of varying training sample size due to the different matched samples and the remotion of noisy values influenced further the error evaluation. 

Averages showed that OLI performs better for Cuitzeo ($\bar{R}$^2^ = 0.55) than the other lakes, behaving the worst in Yuriria $\bar{R}$^2^ = 0.21). Its best prediction is obtained for Turbidity ($\bar{R}$^2^ = 0.42). OLCI performs better in Cuitzeo ($\bar{R}$^2^ = 0.67) and Yuriria ($\bar{R}$^2^ = 0.52) and better predicts Turbidity ($\bar{R}$^2^ = 0.69) and SDD ($\bar{R}$^2^ = 0.50). MSI has on average higher performances due to a low number of training samples in Cuitzeo, Yuriria, and Catemaco, due to poor image coverage in sampling dates and cloud coverage in the few matching images (Table A3). The comparison is feasible only with Chapala where its performance is similar ($\bar{R}$^2^ = 0.45) and Pátzcuaro where it is the poorest ($\bar{R}$^2^ = 0.21). An easier comparison comes from analyzing the model performances by water parameter. Turbidity resulted in a higher performance ($\bar{R}$^2^ = 0.71) with also relatively good Chl-a and SDD ($\bar{R}$^2^ = 0.64 and $\bar{R}$^2^ = 0.61).

From the water parameters, TSM showed to be the most challenging parameter to model as seen in the poor performance in terms of error metrics for all sensors (OLI: $\bar{R}$^2^ = 0.42, OLCI: $\bar{R}$^2^ = 0.35, MSI: $\bar{R}$^2^ = 0.48). Additionally, Chl-a was also poorly correlated for OLI and OLCI (OLI: $\bar{R}$^2^ = 0.18, OLCI: $\bar{R}$^2^ = 0.36). Turbidity models displayed the best performances for Chapala, Cuitzeo Pátzcuaro, and Yuriria, shown in Table A4, Table A5 and Table A6. Individually, no model could retrieve a good correlation for TSM in Yuriria in any sensor (OLI: *R*^2^ = 0.19, OLCI: *R*^2^ = 0.34, MSI: *R*^2^ = 0.11), Chl-a in Pátzcuaro (OLI: *R*^2^ = 0.14, OLCI: *R*^2^ = 0.38, MSI: *R*^2^ = 0.21) in Table A4, Table A5 and Table A6. For the 5 lakes and 4 parameters to the model, ELM occurrence (37 times) for better performance was the highest among all sensors, followed by LR (15 times) and SVR (8 times) (Table 4).

ELM was better suited for OLCI data, and LR was useful in few samples (MSI). SVR showed not to be suited for MSI, most likely because of the combination of limited data and better suitability of ELM and LR (Table A4, Table A5 and Table A6).

### 4.3. Spatial Patterns from Sentinel-3 OLCI WFR and Estimated Parameters

**Figure 7:**
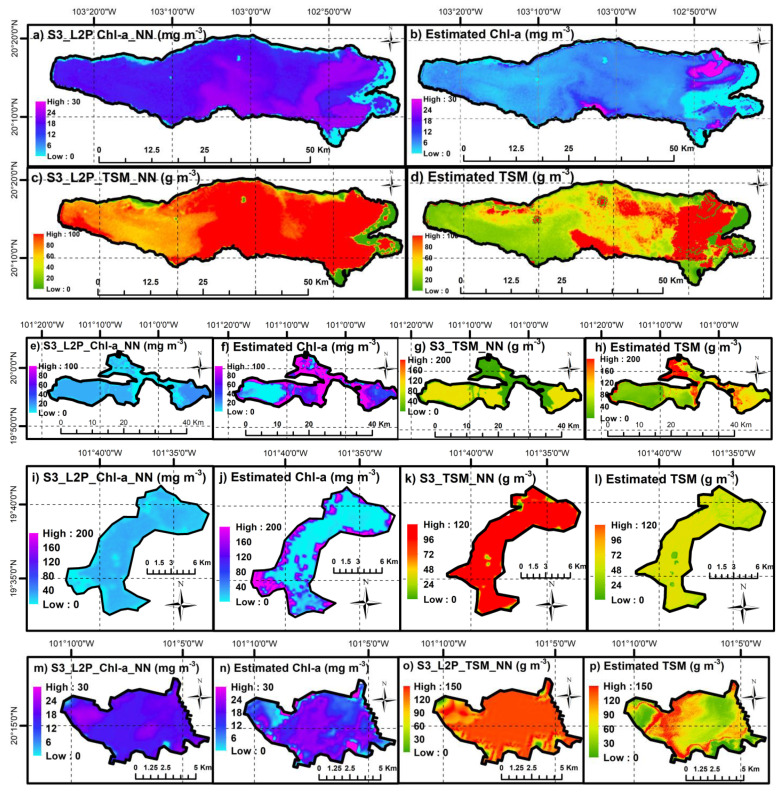

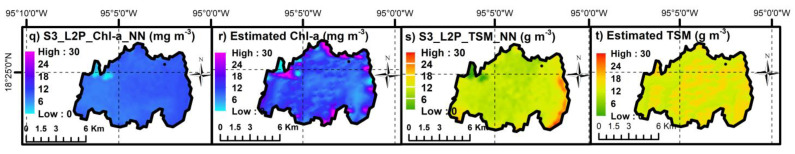
Comparison of Chlorophyll-a (Chl-a) and total suspended matter (TSM) estimations by OLCI WFR products and developed the models in this study for all lakes.

Locally calibrated models were used to produce spatial distribution maps of Chl-a and TSM. These maps were also compared against the OLCI WFR retrievals. The map product of the modeling, regardless of the uncertainties associated with its empirical nature, contribute to a higher understanding of the spatial distribution of water parameters in comparison with being based on sampling stations. The complete maps for all lakes are shown in Figure 7. For display purposes, a random image was selected for its usefulness for visual analysis on every lake. 

Derived maps suggest different interpretations in terms of magnitude and distribution of the parameters between both types of derivations. In Lake Chapala, Chl-a concentrations from OLCI products are generally overestimated, which is visible in most of the lake surface where no significant changes are observed. The east part of the lake shows more variations in the quality in agreement with the location of river discharges discussed in Section 2. In agreement with the previous histograms, OLCI TSM presents fixed values near 100 g m^−3^ mainly in the east part of the lake (Figure 7a–d). Similarly, Lake Cuitzeo varies from homogeneous distribution of Chl-a and TSM from OLCI products to a higher variability depending on the section of the lake with apparent underestimation from OLCI (Figure 7e). Lake Pátzcuaro displayed similitudes between OLCI and modelled Chl-a, with visible differences only near the shores of the lake, especially in the southwest (Figure 7i,j). OLCI TSM for Lake Pátzcuaro also shows overestimation compared with modelled TSM (Figure 7k,l). Lake Yuriria shows the different spatial distribution of both Chl-a and TSM. However, the patterns of river discharges carrying suspended solids (Figure 7p) are clearly distinguishable only in the maps derived from the models.

For Lake Catemaco (Figure 7q) and Lake Pátzcuaro (Figure 7i), lower differences are observable for Chl-a OLCI products and maps from models. However, TSM showed higher variability in Pátzcuaro from 85 g m^−3^ for OLCI products to lower values of 50 g m^−3^ in the calibrated models. As a general observation, more refined details and distinguishable patterns can be discernible in the maps from the trained models; for Chl-a estimations, for example, regions with higher Chl-a content seem to be revealed in areas where primary productivity may be expected to occur as in river discharge or nutrients deposit. Additionally, further characteristics of each lake not studied in this work, such as wind direction, bathymetry, hydrodynamic and morphological features, might induce strong influence in the distribution and magnitude of both parameters.

## 5. Discussion

### 5.1. OLCI Water Products 

Significant limitations exist when integrating remote sensing available products to support the monitoring system in Mexico. A substantial mismatch between OLCI WFR and field measurements was found (Figure 3). OLCI product tends to overestimate Chl-a and TSM values. A deeper analysis on each lake individually displayed little correspondence, except for Chl-a and TSM in Lake Yuriria (Figure A1). Furthermore, an analysis of the spatial distribution of such parameters based on single images showed that patterns regarding the path and concentration of these parameters are difficult to detect via OLCI WFR layers. Likewise, the surface of the lakes appears to be highly homogeneous, while significant spatial variations were identified via local modeling (Figure 7). It is vital to notice that OLCI L2 algal pigment concentration is conceived for ocean products, and its range varies from 0.01 to 100 mg m^−3^ in Case 2 waters. The lakes in this study had lower concentrations, particularly Lake Chapala (Table A1) and therefore an overestimation of Chl-a is not surprising. A similar situation occurs with TSM. These findings suggest that, to rely only on these products to support monitoring program tasks is unfeasible, and therefore there is the need for locally trained models. Even with the disagreement between OLCI WFR and field data from the RNMCA, OLCI L1C products were found to be among the most reasonable sensors to use when monitoring big enough inland waters in the region.

### 5.2. Comparison of Sensors

OLI has outstanding features in terms of spatial resolution and adequate preprocessing routines. In this study, these features contributed to keeping spectral information from proximal stations to water shores. Additionally, its launch in 2013 increased the number of matched images with RNMCA substantially. Its application is further endorsed by years of legacy from the Landsat constellation and its perpetual presence in research of remote sensing of inland waters [18,20,79,80,81]. Therefore, it was expected to retrieve better results from models trained with its radiometric data, although essential restrictions constrained its application. The temporal resolution (16 days) limits the number of matches with the existing field data. Despite its longevity, the retrieved datasets were of similar size to OLCI and the large archive advantage was not compelling to gain a privileged place as the best sensor (Table A4 and Table A5). This limitation was already appointed by Mandanici [82] who compared OLI and MSI, finding limitations in OLI temporal resolution for continuous water quality monitoring. Furthermore, as OLI is designed to observe the main features in earth and not water resources; except for the NIR-band, the spectral amplitude and spectral coverage of the other bands in the VIS region measured by OLI sensor represent a common limitation to carry out specific studies related to the water quality assessment. In other words, OLI is not designed to observe characteristics and features of important parameters involving algae blooms such as Chl-a, commonly used as a proxy of the trophic state of inland waters. 

In the sense of the above, the spectral resolution of OLCI is higher (21 bands) than OLI (11 bands), and the bands are located in relevant regions of the spectra for water quality monitoring. Specifically, wavelengths at 681 nm or at the range 700–710 nm allowed the development of Chl-a based algorithms as the fluorescent line height (FLH) [83] or the maximum chlorophyll index (MCI) [84]. Furthermore, the location of many bands in the Red and NIR regions (b8–12) allowed Turbidity and TSM patterns to be detected. The temporal resolution is reinforced with both S3-A and S3-B, which could have potentiated the found matching data. Unfortunately, the availability of Sentinel-3B was limited when investigating matching dates of the RNMCA, and the data remained limited to S3-A except for a couple of S3-B images. The major limiting factor was possibly the spatial resolution when working with stations close to shores, as some existed in the RNMCA. Due to the size of a single pixel (300 × 300 m) which averages the shore radiances with the ones of adjacent water, such stations were rejected and data availability constrained. If considered, these stations could lead to adjacency error (AE) which would add further uncertainty to the models. For this study and considering the target lakes as the biggest in the country, the resolution of OLCI did not represent a limiting factor as enough pixels were constantly retrieved from the surface of the lakes. However, if OLCI is intended to be used in routinary monitoring tasks, the resolution will likely be a program for small inland waters. It was recently demonstrated that available atmospheric correction procedures, including the C2RCC, for OLCI have difficulties when removing atmospheric disturbances over small inland waters [85]. This is particularly challenging when there are many small lakes as in central Mexico [32], and no radiometric data is measured as part of the RNMCA routines. To improve the methodology applied here, a more comprehensive evaluation needs to analyze different AC or errors associated to different radiometric data as TOA radiances. In terms of model performance from OLCI data, the error metrics (Table 3 and Table 4) even when far from perfect fitting, showed reasonable performances with enough data in most of the lakes and parameters, which may also indicate a good generalization ability.

MSI combines high spatial and temporal resolution due to the availability of both S2-A and S2-B, and expectations with its data were high. Unfortunately, coverage of MSI for the sampling dates and regions of the lakes analyzed in this study was limited and only some images matched with the RNMCA (Table A3). Furthermore, cloud coverage also impeded the processing of some of those few images. This resulted in a limited number of matched points, especially for Lakes Cuitzeo, Yuriria, and Catemaco (Table A6). These factors played an important role. and models using MSI data are likely to lack the generalization ability out of the range of the limited training data. Moreover, similarly to OLI, its design is based on terrestrial and vegetation applications. Therefore current AC processes for S2 are still a matter of research to further validate the quality of the corrected radiometric data over different inland waters [27].

### 5.3. Data Analytics and Machine Learning Modeling

The data analytics routine demonstrated to be useful, as seen in the developed models using spectral and RNMCA data. As seen in the exploratory data, not all the bands or band ratios of the sensors strongly correlated with the water quality parameters (Figure 3). This was corroborated with poor performances from the models in specific lakes or parameters (Table A4, Table A5 and Table A6). However, good agreements were also found and models with strong correlations were developed for each sensor, particularly for turbidity, which exhibited constant high performance in every lake. The usage of shallow algorithms as LR and SVR is shown to be a good base for modeling tasks in inland waters, since they provide straightforward results that can serve as a proxy for test modeling.

Regarding ELM, it is a state-of-the-art shallow algorithm that is in constant development and starts to be applied in evaluations of remote sensing of inland waters [23,65]. Furthermore, the usage of ELM in this study resulted in being useful to develop more accurate models in certain cases. Since it is expected that acquisitions from the RNMCA will continue, further validation and calibration should complement and adapt the models developed here and open new doors for more accurate algorithms. This is especially valid for the use in research of machine learning models, which are in constant change and evolution. Additionally, the evaluation of machine learning models against approaches as empirical-based or bio-optical models is important to further understand the more suitable methodology for remote sensing of inland waters. The promising performance of machine learning models was found in this study. To go further, alternatives such as Deep Learning has already been pointed out in recent research [65,66] as an adequate methodology to deal with the challenges of working with enormous amounts of data storage (as satellite images could be) and water quality datasets. In addition, although different approaches may lead to more robust or generalizable models, its application may be complex and could require large amounts of in-situ data for calibration [63]. These requirements greatly restrict the application of semi-analytical models, favoring empirical approaches. Here is where machine learning offers a good balance between computational power and straightforward application. In this study, machine learning approaches were a useful methodology applied to consistently evaluate the available data from RNMCA. The results suggest that these models are adequate approaches to support the monitoring tasks in emerging economies like Mexico. 

The challenges in the application of machine learning approaches are primarily due to the calibration of the hyperparameters. LR has the advantage of not requiring this calibration, but the relationships between water quality parameters and radiometric data are often not linear. Therefore, the importance of a good calibration of SVR and ELM is to reveal non-linear patterns or non-normally distributed data as water quality parameters. Regarding SVR, its application resulted in being straightforward due to the considered hyperparameters calibrated. Nevertheless, if a broad spectra of hyperparameters are considered, this can lead to long training time. To reduce the computing time, the usage of known kernels and particular values for the regularization parameter (C) and the kernel coefficient (γ) based on powers of 10 resulted in a comprehensive grid that covered a wide range of possible combinations. The case of ELM was further challenging due to the many activation functions available for neural networks and the large number of hidden neurons that can be taken. Ideally, considerations regarding the convergence speed of the ELM with the activation function or perseverance of normalization. These were evaluated by considering training times and normalization of the data before training. However, activation functions did not influence training times as much in Caret using the RNMCA and radiometric data, but more challenging was determining the number of hidden neurons in the hidden layer. From Huang et al. [67], it is known that the generalization performance is stable on a wide range of number of the hidden neurons. Therefore, a range of values based on powers of 10 resulted again in an effective way to find variations in the ELM performance. A small number of neurons (≈10) retrieved results tending to the mean of the training samples. A number of neurons greater than 15,000 or 20,000, together with training data of n > 100 and a LOOCV, resulted in hours of training and minimum improvement of the error metrics, which is not the optimal performance of an ELM. The range of hidden neurons in this study was found to be optimally varied between 1000 and 10,000. A further sensitivity analysis of the number of neurons to better set a range of the ideal number of neurons of an ELM using remote sensing and water quality data is recommended. In addition, it is important to consider that there are additional model capability limitations from radiometric data product of time delay in matching satellite and water quality data [33] and the limited amount of matching data, which leads to small datasets for model training. To face these challenges, we exclude region/pixels which may be corrupted by clouds or adjacency effects, and therefore possible uncertainties and the model routines are evaluated with strict LOOCV. However, this study used data from a single atmospheric correction and did not evaluate further possibilities nor estimate the uncertainties.

### 5.4. Integration of Remote Sensing and the RNMCA

The performed analysis of the selected parameters using OLI, OLCI, and MSI stresses the complications of retrieving accurate estimations of water quality in the region using the available data. From the investigated parameters, turbidity is the parameter better positioned to be estimated using optical and RNMCA data. Different and varied factors may enhance difficulties in estimations. Limitations regarding the nature of the RNMCA and the modeling itself were present in the development of this work and pose challenges for future integration of remote sensing and water quality data.

The current state of the RNMCA data and its acquisitions routines is independent of the satellite acquisitions. Consequently, a lack of synchronization between the field campaigns to measure in-situ data and the satellite overpasses leads to the rejection of data for being too sparse in time (Table A3). Similarly, only some fixed stations on each lake are regularly sampled in field campaigns. For specific dates, there are no measurements of all the stations, most likely subject to different priorities, and limiting the availability of data. Moreover, in-situ radiometric measurements are not routinely measured, impeding validation of the reflectance of the sensor and identification of the optical water types or consideration of further modeling approaches. In addition, the frequency of field campaigns limiting because it was observed to be limited to a couple of times every year or in single campaigns spread over several days. This detaches samples from the date of satellite acquisition, leading to reduction of usable data. This situation affected to a minor degree to Lake Chapala or Lake Catemaco, but it was observed more frequently in Lake Yuriria and Lake Catemaco. In the studied lakes, some sampling stations are located near the shores, which leads to adjacency effects on the acquired radiometric energy in that location and induces errors in the modeling process. Therefore, in this study, these stations were rejected due to its proximity to the land. Access to the data is also of major importance. For instance, the complete dataset of all the measured parameters in the last decade was available only until late 2019. Before this, the available information was limited to information regarding biological oxygen demand (BOD_5_), chemical oxygen demand (COD), and total suspended matters (TSM). Since the water quality information from the RNMCA focused on displaying the results rather than its acquisition and usage, application and research from it were limited. By the beginning of 2020, the increase of data availability and the possibility to use it in an adequate format improved conditions for monitoring research. Currently, this data is also already available in the global freshwater quality database portal (GEMStat), where until the writing of this paper, Mexico leads the amount of data water quality data available for inland waters [86]. 

There are also inherent challenges regarding the methodologies in field campaigns, the use of radiometric data or the wind and temperature conditions in the lakes. From field measurements methodologies described in Section 3.1, we assume that samples collect water from the surface up to a maximal depth of 15–30 cm. For those parameters which require less water volume in sampling, such as turbidity (100 mL), it is likely that the sample is filled at a lower depth than for those that require more, such as TSM or Chl-a (1000 mL), but for all cases the limit is between 15 and 30 cm. This lead us to the assumption that, for Chl-a, turbidity, and TSM, possible discrepancies due to NIR bands are not relevant or relevant in a lower degree Still, the depth of sampling and the effective depth of remote sensing reflectance might not match, which surely add some uncertainties, however, this is a common issue in the remote sensing studies. In our case, this is supported by the fact that turbidity showed the better metrics for all parameters and its models include NIR bands consistently (Table A4 and Table A6) or the use of NIR bands in studies where similar parameters are studied [30] or even at deeper measurements [80,87]. Partial discrepancies due to sampling methods are, however, very likely for SDD. For example, Secchi Disk Depth is based on a subjective measure from the operator, and it reflects the total depth in which the disk is lost from sight, therefore, it is not representing a property of a shallow layer of water directly. Furthermore, the SDD in average is higher than the NIR penetration rate in water of 10 cm (Table A1). In this case, the use of NIR bands may represent a bias in the developed models, which use these bands. Therefore, results for SDD should be taken with caution and not be conclusive. Prevailing winds are capable of generating dispersion phenomena as a result of forcing or friction on the water surface that induces waves, as well as the generation of aerosols due to natural evaporation processes. Additionally, vertical mixing induces movement of waters due to temperature gradients and this may conditionate the satellite measurements. The study areas are located in low wind zones [88], but the wind patterns or prevailing winds should be considered as a challenge for toe reliability of results [89]. Determination of temperature profiles to find mixing areas would also contribute to increase the reliability of the estimations and the determination of the associated uncertainties. The atmospheric correction has several challenges to deal with, as the many variables influencing the radiance spectra used to train the models. Low signal-to-noise-ratio product of the small amount of reflected light in water bodies requires an accurate correction for atmospheric contributions [90]. The predominance of the effect of a specific constituent over another in the water may create the masking effect and avoid estimating the non-dominant constituent in the water with enough confidence [56]. Since turbidity depends strongly on TSM and Chl-a, and given that all the lakes (except lake Catemaco) preserve relative high concentrations of both parameters; turbidity was the parameter better positioned among the investigated parameters. On the other side, radiometric data may be improved through preprocessing. An additional glint-correction on the image may help to improve the response derived from the sub-surface water layer, retrieving a more reliable water-leaving radiance this may help to diminish the dispersion effect produced by the reflection of the wind-waves, aerosols associated to natural evaporation in the air-water layer.

To include remote sensing into the RNMCA routines, it is important to understand the obstacles of integration. One of the main reasons is the lack of awareness or expertise from local managers of remote sensing techniques. Schaeffer et al. [31] assessed this situation in a local study in the US. According to their findings, local managers commonly lack the knowledge to interpret and use the technical descriptions of remote sensing and modeling techniques to retrieve water parameters. Therefore dialog between researchers and resource managers is essential and applications that foster the embracing of remote sensing are necessary to show its full potential continuously. After the limitations described in this work, it is clear that requirements for the applications of remote sensing approaches should be considered when designing sampling campaigns. For example, the field campaigns may consider synchronization with satellite acquisitions after a revision of the most adequate sensor and considering the spatial and temporal requirements. Scales of days are needed and resolutions below 300 m and 30 m are adequate for big and small lakes, respectively. The number of field campaigns and sampling stations are equally important. A minimum of four field campaigns per year is recommended for the United Nations Environment Program (UNEP) [2], one for each season. In Catemaco or Yuriria, the fourth and fifth biggest lakes in the country only have five and four stations, respectively. The loss of one or two stations are due to shore proximity or incomplete sampling limits data availability. Furthermore, the inclusion of in-situ radiometric data measurements to validate the satellite’s sensors reflectance spectra and further classify the optical water types. For this, upwelling radiance from water and downwelling sky radiance are key parameters. Routines of calibration should then be applied to normalize this data to downwelling irradiance. The derivation of remote sensing reflectance can be then done through known procedures [91].

The benefits for water managers are well known. Costs regarding time consumption in field campaigns and human resources can be mitigated, and the enhancement of spatial and temporal information is highly significant compared to field campaigns alone. The recommendation for integration is already suggested by the UNEP, where the advice is to schedule and adapt the field campaigns considering the use of remote sensing for water monitoring [2]. The initial step could be the most challenging since it will require an assumption from the highest level of water managers to direct how the field campaigns are being taken. Meanwhile, the data from the RNMCA are highly valuable and set the basis for an extensive network for water quality monitoring. The identification of fixed stations, the high variety of water parameters measured (around 30 in the complete dataset), and its endurance through the years will likely allow high-quality and comprehensive data to be available for practical and research applications. Besides, it should be clear that a hypothetical integration of remote sensing into the design of management routines will be in continuous evolution. It is expected that the routines are adapted with the availability of future sensors, which can incorporate better features suited for inland water quality monitoring. Furthermore, resources such as cloud computing should be taken into account to develop large applications that process great amounts of data as a monitoring water system at a national level or satellite imagery. Cloud computing infrastructure and applications based on AI may facilitate water quality monitoring through access to NASA, USGS, and ESA archives.

## 6. Conclusions

This work focuses on the implications of the lack of integration of remote sensing in water quality monitoring routines, taking as a study case the existing monitoring system (RNMCA) of the five biggest lakes in the country. Available ready-to-use products from Sentinel-3, OLCI WFR, tend to overestimate or underestimate field values for Chl-a and TSM, and validation results indicate a need for alternative approaches based on field data. Through a routine based on data analytics and machine learning algorithms, Landsat-8 OLI, Sentinel-3 OLCI, and Sentinel-2 MSI sensors were studied to investigate their suitability to monitor water quality in the region. ELM resulted in the most used methodology for modeling followed by LR and SVR. The results ranged widely in performance, from weak to strong relationships. From the three sensors, Sentinel-3 OLCI showed a moderate better performance over Landsat-8 and Sentinel-2 MSI for these study areas with good results for turbidity and moderate correlations for Chl-a, TSM, and SDD. The current state of the data and the fact that the RNMCA is developed independently and without considering remote sensing techniques placed different difficulties in the processing methodology and led to considerable losses of in-situ data. It is recommended to study the local needs and available in-situ data when choosing a sensor for monitoring water quality or design routinely field campaigns based on the acquisition calendar of only one sensor. Due to their design, it is not possible to select a sensor for monitoring inland waters. To magnify their use and full potential, synergistic applications should be developed to combine the strengths of various sensors and mitigate their limitations. This work contributes to creating awareness of the misuse and absent application of remote sensing by water managers in emerging economies for water monitoring routines. The progress of remote sensing for inland waters is bound to its usage to improve existing monitoring tasks, which will impact management and protection of water resources. 

## Figures and Tables

**Table 1:** Characteristics of the optical sensors used in this study.

Satellite	Temporal Resolution (Days)	Spatial Resolution (m)	Launched	Spectral Bands
Landsat-8 OLI	16	30	2013	11
Sentinel-3A and 3B	2–3	300	3A, 2016; 3B, 2018	21
Sentinel-2A and 2B	5	10 and 20	2A, 2015; 2B, 2017	13

**Table 2:** Average coefficient of determination (R^2^) of the developed models by sensor and lake together with the average number of samples (n). Operational Land Imager (OLI) (left), Ocean and Land Color Instrument (OLCI) (center), and Multi Spectral Instrument (MSI) (right).

Lake	OLI		OLCI		MSI	
	R^2^	n	R^2^	n	R^2^	n
Chapala	0.37	141	0.45	75	0.45	44
Cuitzeo	0.55	23	0.67	19	0.90	7
Pátzcuaro	0.37	43	0.42	31	0.21	39
Yuriria	0.21	16	0.52	17	0.60	11
Catemaco	0.27	17	0.32	17	0.88	7

**Table 3:** Average error metrics of the trained models by sensor and water parameter. Error metrics of all lakes are included on each water parameters RMSE: Chla in mg m^−3^, Turbidity in NTU, TSM in g m^−3^, SDD in m. The number of samples is also displayed on average.

Parameter	OLI				OLCI				MSI			
	R^2^	RMSE	MAE	n	R^2^	RMSE	MAE	n	R^2^	RMSE	MAE	n
Chl-a (mg m^−3^)	0.18	19.99	13.81	40	0.36	21.27	8.86	32	0.64	8.47	6.23	20
Turbidity (NTU)	0.48	35.23	23.31	51	0.69	17.80	30.99	30	0.71	17.24	47.40	22
TSM (g m^−3^)	0.42	107.31	40.37	49	0.35	33.12	28.10	32	0.48	118.21	24.70	23
SDD (m)	0.33	0.08	0.05	52	0.50	0.26	0.18	32	0.61	0.45	0.36	23

**Table 4:** Model occurrence in every sensor as a result of better predictive capabilities.

Model	OLI	OLCI	MSI	Total
ELM	10	14	13	37
SVR	5	3	-	8
LR	5	3	7	15
Total	20	20	20	60

## Data Availability

Not applicable.

## Appendix A

**Table A1:** Descriptive statistics of the RNMCA dataset.

Lake	Stations	Parameter	Count	Mean	St. Dev.	Min	25%	50%	75%	Max
Chapala	26	Chl-a (mg m^−3^)	307	8.3	21.7	0.0	0.1	0.9	8.3	218.4
		Turbidity (NTU)	387	36.2	51.1	0.3	18.0	24.0	34.0	750.0
		TSM (mg L^−1^)	388	49.5	61.4	10.0	18.0	30.1	52.0	475.0
		SDD (m)	388	0.4	0.2	0.1	0.3	0.4	0.5	2.3
Cuitzeo	9	Chl-a (mg m^−3^)	116	35.7	41.7	0.1	8.0	20.3	50.3	247.5
		Turbidity (NTU)	120	181.4	191.5	2.5	52.8	122.5	238.5	1176.0
		TSM (mg L^−1^)	118	152.4	159.9	10.0	56.3	96.5	170.0	790.0
		SDD (m)	121	0.1	0.1	0.0	0.1	0.1	0.2	0.7
Pátzcuaro	17	Chl-a (mg m^−3^)	266	27.3	39.9	0.4	7.6	15.8	26.2	395.4
		Turbidity (NTU)	268	92.2	43.6	4.5	62.2	87.3	115.2	302.7
		TSM (mg L^−1^)	268	47.6	36.1	6.0	20.0	38.5	64.0	250.0
		SDD (m)	268	0.2	0.1	0.0	0.2	0.2	0.2	0.5
Yuriria	6	Chl-a (mg m^−3^)	75	21.4	20.5	0.1	7.3	13.5	30.7	91.3
		Turbidity (NTU)	76	92.3	45.6	26.4	65.9	82.5	115.5	262.7
		TSM (mg L^−1^)	76	65.3	69.7	8.0	21.5	41.0	73.3	333.0
		SDD (m)	76	0.2	0.1	0.1	0.2	0.2	0.2	0.5
Catemaco	4	Chl-a (mg m^−3^)	57	26.1	62.8	0.0	0.1	10.1	28.3	345.8
		Turbidity (NTU)	58	4.9	4.2	1.0	3.1	3.8	5.1	31.3
		TSM (mg L^−1^)	58	17.4	14.8	10.0	10.0	12.5	17.5	90.0
		SDD (m)	57	0.8	1.3	0.2	0.5	0.6	0.8	10.0

**Table A2:** Summary of the bands used in this study.

OLI	b1	b2	b3	b4	b5	b6	b7					
Wavelength (nm)	0.43–0.45	0.45–0.51	0.53–0.59	0.64–0.67	0.85–0.88	1.57–1.65	2.11–2.29					
Resolution (m)	30	30	30	30	30	30	30					
OLCI	Oa01	Oa02	Oa03	Oa04	Oa05	Oa06	Oa07	Oa08	Oa09	Oa10	Oa11	Oa12
Center (nm)	400	412.5	442.5	490	510	560	620	665	673.75	681.25	708.75	753.75
Resolution (m)	30	30	30	30	30	30	30	15	30	100	100	
MSI	b1	b2	b3	b4	b5	b6	b7	b8	b8a			
Center (nm)	442.7	492.4	559.8	664.6	704.1	740.5	782.8	832.8	864.7			
Resolution (m)	60	10	10	10	20	20	20	10	20			

**Table A3:** Matching satellite acquisitions and RNMCA sampling dates. Complete table with images ID is provided in the complementary material.

Sensor	Lake	Matching Images	Total
OLI	Chapala	13	
	Cuitzeo	11	
	Pátzcuaro	4	
	Yuriria	4	
	Catemaco	9	41
OLCI	Chapala	12	
	Cuitzeo	13	
	Pátzcuaro	6	
	Yuriria	9	
	Catemaco	7	47
MSI	Chapala	10	
	Cuitzeo	4	
	Pátzcuaro	5	
	Yuriria	9	
	Catemaco	3	31

**Table A4:** OLI Model validation and predictive capability results for all datasets including coefficient of determination (R^2^), root mean square error (RMSE), mean absolute error (MAE), and the number of samples (n).

Sensor	Lake	Parameter	Model	Hyperparameters	Bands Used	R^2^	RMSE	MAE	n
OLI	Chapala								
		Chl-a	ELM	af = hardlims, hn = 10,000	All	0.38	4.63	3.22	100
		Turbidity	LR	-	b2, b4, b5, b6	0.66	20.17	8.80	154
		TSM	ELM	af = hardlims, hn = 10,000	All	0.21	417.07	115.89	154
		SDD	SVR	C = 100, γ = 0.01, k = sigmoid	logb1, logb2, logb4, logb5, logb6, logb7	0.30	0.11	0.08	154
	Cuitzeo								
		Chl-a	ELM	af = hardlim, hn = 150	All	0.13	34.55	20.38	22
		Turbidity	LR	-	b2, b5, b6, b7	0.74	97.64	64.38	22
		TSM	ELM	af = purelin, hn, hn = 15,000	All	0.91	60.86	41.18	14
		SDD	SVR	C: 10, γ = 5, k = sigmoid	logb4, logb5, logb6, logb7	0.41	0.1	0.06	33
	Pátzcuaro								
		Chl-a	ELM	af = hardlims, hn: 1000	All	0.14	34.98	25.43	43
		Turbidity	SVR	C = 500, γ = 5	All	0.56	27.75	19.21	43
		TSM	SVR	C = 1000, γ = 500	b2, b4	0.38	19.9	14.85	43
		SDD	SVR	C = 0.5, γ = 500	b3, b4, b5	0.42	0.04	0.03	43
	Yuriria								
		Chl-a	ELM	af = hardlims hn: 5000	All	0.11	15.88	12.56	16
		Turbidity	LR	-	b1, b3, b5, b7	0.32	21.42	18.23	16
		TSM	SVR	C = 0.5, γ = 1 × 10^−5^, k = linear	logb5	0.19	20.83	15.73	16
		SDD	LR	-	b1, b4	0.22	0.046	0.031	16
	Catemaco								
		Chl-a	ELM	af = hardlim, hn = 10,000	All	0.15	9.90	7.47	18
		Turbidity	ELM	af = hardlims, hn = 10,000	All	0.12	9.16	5.91	18
		TSM	ELM	af = hardlim, hn = 8000	All	0.42	17.87	14.20	16
		SDD	ELM	af = hardlims, hn = 100	All	0.40	0.09	0.07	16

af: Activation function, hn: Number of hidden neurons, C: Regularization parameter, γ: Gamma for SVR, k: kernel.

**Table A5:** OLCI Model validation and predictive capability results for all datasets including coefficient of determination (R^2^), root mean square error (RMSE), mean absolute error (MAE), and the number of samples (n).

Satellite	Lake	Parameter	Model	Hyperparameters	Bands Used	R^2^	RMSE	MAE	n
OLCI									
	Chapala								
		Chl-a	ELM	af = hardlims, hn = 500	All	0.25	3.07	1.92	77
		Turbidity	ELM	af = hardlims, hn = 500	-	0.67	6.08	4.47	72
		TSM	ELM	af = hardlims, hn = 500	All	0.20	14.47	9.63	74
		SDD	SVR	C = 10, γ = 0.1, k = ‘sigmoid’	b8	0.67	0.06	0.05	77
	Cuitzeo								
		Chl-a	ELM	af = purelin, hn = 10	All	0.47	59.94	16.20	20
		Turbidity	ELM	af = harlim, hn = 10,000	All	0.82	44.99	35.75	13
		TSM	LR	-	b1, b10, b11, b3, b4, b5, b6, b7, b9	0.58	77.83	55.09	21
		SDD	ELM	af = poslin, hn = 10,000	All	0.82	0.04	0.12	21
	Pátzcuaro								
		Chl-a	ELM	af = hardlims, hn = 10,000	All	0.38	26.74	16.19	31
		Turbidity	LR	-	b7, b8	0.77	14.08	10.62	31
		TSM	SVR	C = 500, γ = 10	logb1, logb2, logb3, logb4, logb5, logb6, logb7	0.36	20.32	15.86	31
		SDD	ELM	af = purelin, hn = 5	All	0.18	0.02	0.02	29
	Yuriria								
		Chl-a	SVR	C = 500, gamma = 1, k = ‘rbf’	logb1, logb2, logb3, logb4, logb5	0.32	8.61	4.373	17
		Turbidity	ELM	af = tribas, hn = 15,000	All	0.83	23.32	100.85	18
		TSM	ELM	af = tansig, hn = 10	All	0.34	51.12	48.06	17
		SDD	ELM	af = purelin, hn = 50	All	0.60	0.11	0.25	17
	Catemaco								
		Chl-a	LR	-	b1, b4, b6, b7	0.38	8.01	5.63	17
		Turbidity	ELM	af = harlim, hn = 10,000	All	0.36	0.54	3.27	16
		TSM	ELM	af = harlim, hn = 1000	All	0.26	1.86	11.89	17
		SDD	ELM	af = tribas, hn = 12,500	All	0.26	1.04	0.45	16

af: Activation function, hn: Number of hidden neurons, C: Regularization parameter, γ: Gamma for SVR, k: kernel.

**Table A6:** MSI Model validation and predictive capability results for all datasets including coefficient of determination (R^2^), root mean square error (RMSE), mean absolute error (MAE), and the number of samples (n).

Satellite	Lake	Parameter	Model	Hyperparameters	Bands Used	R^2^	RMSE	MAE	n
MSI									
	Chapala								
		Chl-a	ELM	af = hardlims, hn = 10,000	All	0.24	2.68	1.51	26
		Turbidity	ELM	af = hardlims, hn = 10,000	All	0.60	7.37	25.62	47
		TSM	ELM	af = satlins, hn = 15,000	All	0.43	45.08	38.69	52
		SDD	LR	-	logb3, logb4, logb6, logb7, logb8	0.53	0.08	0.06	52
	Cuitzeo								
		Chl-a	LR	-	logb3, logb6, logb1, logb7, logb8	0.98	0.92	0.76	7
		Turbidity	LR	-	b1, b2, b3, b4, b6	0.91	18.53	16.35	7
		TSM	LR	-	b1, b2, b4, b6, b7	0.94	13.4	9.81	7
		SDD	ELM	af = hardlim, hn = 5000	All	0.78	0.07	0.07	7
	Pátzcuaro								
		Chl-a	ELM	af = radbas, hn = 500	All	0.21	25.91	10.15	47
		Turbidity	ELM	af = tribas 5000	All	0.36	44.90	104.14	36
		TSM	ELM	af = poslin, hn = 10,000	All	0.14	423.63	35.25	36
		SDD	LR	-	logb6, logb7	0.15	1.96	1.6	36
	Yuriria								
		Chl-a	ELM	af = hardlims, hn = 10,000	All	0.81	4.85	13.57	11
		Turbidity	ELM	af = poslin, hn = 10,000	All	0.82	14.63	90.41	11
		TSM	ELM	af = satlins, hn = 1500	All	0.11	107.57	38.64	11
		SDD	LR	-	logb1, logb3, logb4, logb5, logb6, logb7, logb8	0.68	0.07	0.05	11
	Catemaco								
		Chl-a	ELM	af = hardlims, hn = 15,000	All	0.95	7.97	5.14	7
		Turbidity	ELM	af = hardlim, hn = 1000	All	0.85	0.79	0.50	7
		TSM	ELM	af = poslin, hn = 1000	All	0.81	1.34	1.09	7
		SDD	LR	-	logb1, logb2, logb5, logb6, logb7	0.91	0.09	0.04	7

af: Activation function, hn: Number of hidden neurons, C: Regularization parameter, γ: Gamma for SVR, k: kernel.

## References

[1] Morris B.L., Lawrence A.R., Chilton P.J., Adams B., Calow R., Klinck B.A. (2003). Groundwater and its susceptibility to degradation: A global assessment of the problems and options for management. Early Warning and Assessment Report Series.

[2] UNEP (2016). A Snapshot of the World’s Water Quality: Towards a Global Assessment.

[3] (2014). Iagua, Brazil launches the National Water Quality Monitoring Network. https://www.iagua.es/noticias/brasil/14/03/25/brasil-lanza-la-red-nacional-de-monitoreo-de-la-calidad-del-agua-47365.

[4] Ambiental R.F.D.M. (2021). Red de Monitoreo Ambiental (Agua). https://redfema.ambiente.gob.ar/monitor/agua.

[5] Meza A.F. (2009). Control de calidad de las aguas en Chile. Tierra Adentro.

[6] Giardino C., Bresciani M., Cazzaniga I., Schenk K., Rieger P., Braga F., Matta E., Brando V.E. (2014). Evaluation of multi-resolution satellite sensors for assessing water quality and bottom depth of lake garda. Sensors.

[7] El-Din M.S., Gaber A., Koch M., Ahmed R.S., Bahgat I. (2013). Remote sensing application for water quality assessment in lake timsah, suez canal, Egypt. J. Remote Sens. Technol..

[8] Mark W., Bernard S., Winter K. (2010). Remote sensing of cyanobacteria-dominant algal blooms and water quality parameters in Zeekoevlei, a small hypertrophic lake, using MERIS. Remote Sens. Environ..

[9] Miller R.L.M., McKee B.A. (2004). Using MODIS Terra 250 m imagery to map concentrations of total suspended matter in coastal waters. Remote Sens. Environ..

[10] Kloiber S.M.B., Brezonik P.L., Bauer M.E. (2002). Application of Landsat imagery to regional-scale assessments of lake clarity. Water Res..

[11] Watanabe F.S.Y.A., Alcântara E., Rodrigues T.W.P., Imai N.N., Barbosa C.C.F., Rotta L.H.D.S. (2015). Estimation of chlorophyll-a concentration and the trophic state of the barra bonita hydroelectric reservoir using OLI/landsat-8 images. Int. J. Environ. Res. Public Health.

[12] Medina-Cobo M.D., Domínguez J.A., Quesada A., de Hoyos C. (2014). Estimation of cyanobacteria biovolume in water reservoirs by MERIS Sensor. Water Res..

[13] Pereira L.S.F.F.A., Andes L.C., Cox A.L., Ghulam A. (2017). Measuring suspended-sediment concentration and turbidity in the middle mississippi and lower missouri rivers using landsat data. JAWRA J. Am. Water Resour. Assoc..

[14] Topp S.N.P., Pavelsky T.M., Jensen D., Simard M., Ross M.R.V. (2020). Research trends in the use of remote sensing for inland water quality science: Moving towards multidisciplinary applications. Water.

[15] Malthus T.J.H., Hestir E.L., Dekker A.G., Brando V.E. The case for a global inland water quality product. Proceedings of the IEEE International Geoscience and Remote Sensing Symposium.

[16] Hastie T., Tibshirani R., Friedman J. (2009). The Elements of Statistical Learning. Data Mining, Inference, and Prediction.

[17] Arias-Rodriguez L.F., Duan Z., Sepúlveda R., Martinez-Martinez S.I., Disse M. (2020). Monitoring water quality of valle de bravo reservoir, mexico, using entire lifespan of meris data and machine learning approaches. Remote Sens..

[18] Lillesand T.M., Johnso W.L., Deuell R.L., Lindstrom O.M., Meisner D.E. (1983). Use of Landsat data to predict the trophic state of Minnesota lakes. Photogramm. Eng. Remote Sens..

[19] Yosef Z., Yacobi A.G., Mayo M. (1995). Remote sensing of chlorophyll in Lake Kinneret using high spectral-resolution radiometer and Landsat TM: Spectral features of reflectance and algorithm development. J. Plankton Res..

[20] Brezonik P., Menken K.D., Bauer M. (2005). Landsat-based remote sensing of lake water quality characteristics, including chlorophyll and colored dissolved organic matter (CDOM). Lake Reserv. Manag..

[21] Wang F., Han L., Kung H.T., van Arsdale R. (2006). Applications of Landsat-5 TM imagery in assessing and mapping water quality in Reelfoot Lake, Tennessee. Int. J. Remote Sens..

[22] Vermote E., Justice C., Claverie M., Franch B. (2016). Preliminary analysis of the performance of the Landsat 8/OLI land surface reflectance product. Remote Sens. Environ. Manag..

[23] Peterson K.T., Sagan V., Sidike P., Cox A.L., Martinez M. (2018). Suspended sediment concentration estimation from landsat imagery along the lower missouri and middle Mississippi rivers using an extreme learning machine. Remote Sens..

[24] Gholizadeh M.H., Melesse A.M., Reddi L. (2016). A comprehensive review on water quality parameters estimation using remote sensing techniques. Sensors.

[25] Odermatt D., Heege T., Nieke J., Kneubühler M., Itten K. (2008). Water quality monitoring for lake constance with a physically based algorithm for MERIS data. Sensors.

[26] Kratzer S., Brockmann C., Moore G. (2008). Using MERIS full resolution data to monitor coastal waters—A case of study from Himmerfjärden, a fjord-like bay in the northwestern Baltic Sea. Remote Sens. Environ..

[27] Ansper A., Alikas K. (2019). Retrieval of chlorophyll a from sentinel-2 msi data for the European Union water framework directive reporting purposes. Remote Sens..

[28] Buma W.G., Lee S.-I. (2020). Evaluation of sentinel-2 and landsat 8 images for estimating chlorophyll-a concentrations in lake Chad, Africa. Remote Sens..

[29] Tamm O., Tamm T. (2020). Verification of a robust method for sizing and siting the small hydropower run-of-river plant potential by using GIS. Renew. Energy.

[30] Bonansea M., Rodriguez M.C., Pinotti L., Ferrero S. (2015). Using multi-temporal Landsat imagery and linear mixed models for assessing water quality parameters in Río Tercero reservoir (Argentina). Remote Sens. Environ..

[31] Blake A., Schaeffer K.G., Keith D., Lunetta R.C., Conmy R., Gould R.E. (2013). Barriers to adopting satellite remote sensing for water quality management. Int. J. Remote Sens..

[32] Atlas del Agua en México. https://agua.org.mx/wp-content/uploads/2019/04/AAM_2018.pdf.

[33] Otto P., Vallejo-Rodríguez R., Keesstra S., León-Becerril E., de Anda J., Hernández-Mena L., del Real-Olvera J., Díaz-Torres J.d.J. (2020). Time Delay Evaluation on the Water-Leaving Irradiance Retrieved from Empirical Models and Satellite Imagery. Remote Sens..

[34] López-Hernández M., Ramos-Espinosa M.G., Carranza-Fraser Y.G. (2007). Jorge Análisis multimétrico para evaluar contaminación en el río Lerma y lago de Chapala, México. Hidrobiológica.

[35] Membrillo-Abad A.-S., Marco-Antonio T.-V., Alcocer J., Prol-Ledesma R.M., Oseguera L.A., Ruiz-Armenta J.R. (2016). Trophic State Index estimation from remote sensing of lake Chapala. Rev. Mex. Cienc. Geol..

[36] de Anda J., Shear H., Maniak U., Valle P.F.Z. (2004). Solids distribution in lake chapala, Mexico. J. Am. Water Resour. Assoc..

[37] Villalobos-Castañeda B., Alfaro-Cuevas R., Cortés-Martínez R., Martínez-Miranda V., Márquez-Benavides L. (2010). Distribution and partitioning of iron, zinc, and arsenic in surface sediments in the Grande River mouth to Cuitzeo Lake, Mexico. Environ. Monit. Assess..

[38] Mendoza M.E., Bocco G., Bravo M., López G.E., Osterkamp W.R. (2006). Predicting Water-Surface Fluctuation of Continental Lakes: A RS and GIS Based Approach in Central Mexico. Water Resour. Manag..

[39] Mendoza M.E., Granados F.L., Geneletti D., Pérez-Salicrup D.R., Salinas V. (2011). Analyzing land cover and land use change processes at watershed level: A multitemporal study in the Lake Cuitzeo Watershed, Mexico (1975–2003). Appl. Geogr..

[40] Mendoza M.E., Bocco G., López-Granados E., Bravo E.M. (2010). Hydrological implications of land use and land cover change: Spatial analytical approach at regional scale in the closed basin of the Cuitzeo Lake, Michoacan, Mexico. Singap. J. Trop. Geogr..

[41] Soto-Galera E., Paulo-Maya J., López-López E. (1999). Change in fish fauna as indication of aquatic ecosystem condition in río grande de morelia-lago de cuitzeo basin, Mexico. Environ. Manag..

[42] Ramírez-Herrejón J.P., Zambrano L., Mercado-Silva N., Torres-Téllez A., Pineda-García F., Caraveo-Paniño J., Balart E.F. (2014). Long term changes in the fish fauna of Lago de Pátzcuaro in Central Mexico. Lat. Am. J. Aquat. Res..

[43] Osorio-Ocampo S., Macíasa J.L., Pola A., Cardona-Melchor S., Sosa-Ceballos G., Garduño-Monroy V.H. (2018). The eruptive history of the Pátzcuaro Lake area in the Michoacán Guanajuato Volcanic Field, central México: Field mapping, C-14 and 40Ar/39Ar geochronology. J. Volcanol. Geotherm. Res..

[44] Metcalfe S.E., Davies S.J., Braisby J.D., Leng M.J., Newton A.J., Terrett N.L., O’Hara S.L. (2007). Long and short-term change in the Pátzcuaro Basin, central Mexico. Palaeogeogr. Palaeoclimatol. Palaeoecol..

[45] Platt B.J. (2000). Limnologic history of Lago de Pátzcuaro, Michoacán, Mexico for the past 48,000 years: Impacts of climate and man. Palaeogeogr. Palaeoclimatol. Palaeoecol..

[46] O’Hara S.L., Street-Perrott F.A.B., Timothy P. (1993). Accelerated soil erosion around a Mexican highland lake caused by prehispanic agriculture. Nature.

[47] (2004). Ficha Informativa de los Humedales de Ramsar (FIR). https://rsis.ramsar.org/RISapp/files/RISrep/MX1361RIS.pdf?language=en.

[48] Tania E.C., Diza S., Elías J., Eugenia L.L. (2013). Evaluación de la calidad del agua en la Laguna de Yuriria, Guanajuato, México, mediante técnicas multivariadas: Un análisis de valoración para dos épocas 2005, 2009–2010. Rev. Int. Contam. Ambient..

[49] Atlas of the natural, historical and cultural heritage of Veracruz: III Cultural heritage. http://libros.uv.mx/index.php/UV/catalog/book/FC147.

[50] Pérez-Rojas A. (1992). Roberto geomorfología y batimetría del lago de catemaco, Veracruz, México. Anales del Instituto de Ciencias del Mar y Limnología.

[51] Guevara S.L., Javier D., Sánchez-Ríos G. (2004). El Paisaje de la Sierra.

[52] Gutiérrez Q.M.G. (2014). Contribución al Estudio de la Diversidad del Zooplancton en Tres Lagos Tropicales y su Relación con el uso de Suelo en Los Tuxtlas.

[53] Berry J.P., Owen L. (2010). First evidence of “paralytic shellfish toxins” and cylindrospermopsin in a Mexican freshwater system, Lago Catemaco, and apparent bioaccumulation of the toxins in “tegogolo” snails (Pomacea patula catemacensis). Toxicon Off. J. Int. Soc. Toxinol..

[54] García-Aguirre M.C., Román Á., Dirzo R., Ortiz M.A., Eng M.M. (2010). Delineation of biogeomorphic land units across a tropical natural and humanized terrain in Los Tuxtlas, Veracruz, México. Geomorphology.

[55] Chacon-Torres A., Beveridge M. (1992). The application of SPOT multispectral imagery for the assessment of water quality in Lake Pátzcuaro, Mexico. Int. J. Remote Sens..

[56] Giardino C., Brando V.E., Gege P. (2019). Imaging spectrometry of inland and coastal waters: State of the art, achievements and perspectives. Surv. Geophys..

[57] Water Environmental Federation, APHA (2005). Standard Methods for the Examination of Water and Wastewater.

[58] (2001). Análisis de Agua—Determinación de Turbiedad en Aguas Naturales, Residuales y Residuales Tratadas—Método de Prueba. https://www.gob.mx/cms/uploads/attachment/file/166777/NMX-AA-038-SCFI-2001.pdf.

[59] (2015). Análisis de Agua—Medición de Sólidos y Sales Disueltas en Aguas Naturales, Residuales y Residuales Tratadas—Método de Prueba. https://www.gob.mx/cms/uploads/attachment/file/166146/nmx-aa-034-scfi-2015.pdf.

[60] (2015). Análisis de Agua—Criterios Generales Para el Control. de la Calidad de Resultados Analíticos. https://www.gob.mx/cms/uploads/attachment/file/166150/nmx-aa-115-scfi-2015.pdf.

[61] (1993). Norma Oficial Mexicana. Nom 014-ssa1-1993 Procedimientos Sanitarios Para el Muestreo de Agua Para uso y Consumo Humano en Sistemas de Abastecimiento de Agua Publicos y Privados. http://dof.gob.mx/nota_detalle.php?codigo=4801645&fecha=12/11/1993.

[62] Ranghetti M.B.F., Nutini L. (2020). Busetto sen2r: An R toolbox for automatically downloading and preprocessing Sentinel-2 satellite data. Comput. Geosci..

[63] Matthews M.W. (2011). A current review of empirical procedures of remote sensing in inland and near-coastal transitional waters. Int. J. Remote Sens..

[64] Blix K.P.K., Tóth V.R., Eltoft T. (2018). Remote sensing of water quality parameters over lake balaton by using sentinel-3 OLCI. Water.

[65] Kyle T., Peterson V.S.J.J.S. (2020). Deep learning-based water quality estimation and anomaly detection using Landsat-8/Sentinel-2 virtual constellation and cloud computing. GISci. Remote Sens..

[66] Vasit S.K.T.P., Maitiniyazi M., Paheding S., John S., Benjamin A., Samar M.G., Craig A. (2020). Monitoring inland water quality using remote sensing: Potential and limitations of spectral indices, bio-optical simulations, machine learning, and cloud computing. Earth-Sci. Rev..

[67] Guang-Bin H., Chee-Kheong S. (2006). Extreme learning machine: Theory and applications. Neurocomputing.

[68] Mouselimis A.G.L. (2021). Documentation: Package ‘elmNNRcpp’. The Extreme Learning Machine Algorithm. https://github.com/mlampros/elmNNRcpp.

[69] Sun D.Q.Z., Li Y., Shi K., Gong S. (2014). Detection of total phosphorus concentrations of turbid inland waters using a remote sensing method. Water Air Soil Pollut..

[70] Azamathulla H.M., Wu F.C. (2011). Support vector machine approach for longitudinal dispersion coefficients in natural streams. Appl. Soft Comput..

[71] Samui P. (2008). Support vector machine applied to settlement of shallow foundations on cohesionless soils. Comput. Geotech..

[72] Pasolli L.M.F., Blanzieri E. (2010). Gaussian process regression for estimating chlorophyll concentration in subsurface waters from remote sensing data. IEEE Geosci. Remote Sens. Lett..

[73] Verrelst J.M.J., Alonso L., Rivera J.P., Camps-Valls G., Moreno J. (2012). Machine learning regression algorithms for biophysical parameter retrieval: Opportunities for Sentinel-2 and -3. Remote Sens. Environ..

[74] Vapnik V.G., Smola A. (1997). Support Vector Method for Function Approximation, Regression Estimation and Signal Processing.

[75] Kuhn M. (2008). Building predictive models in r using the caret package. J. Stat. Softw..

[76] Mouselimis L. elmNNRcpp: The Extreme Learning Machine Algorithm. R Package Version 1.0.3. https://cran.r-project.org/package=elmNNRcpp.

[77] Gosso A. (2012). elmNN: Implementation of ELM (Extreme Learning Machine) Algorithm for SLFN (Single Hidden Layer Feedforward Neural Networks). https://rdrr.io/cran/elmNN/.

[78] Pedregosa G.V.F., Gramfort A., Vincent M., Bertrand T. (2011). Scikit-learn: Machine Learning in Python. J. Mach. Learn. Res..

[79] Zilioli E.B.P. (1997). The satellite derived optical information for the comparative assessment of lacustrine water quality. Sci. Total Environ..

[80] Han L.J.K.J. (2005). Estimating and mapping chlorophyll-a concentration in Pensacola Bay, Florida using Landsat ETM+ data. Int. J. Remote Sens..

[81] Papoutsa C.R.A., Toulios L., Hadjimitsis D.G. (2014). Defining the Landsat TM/ETM+ and chris/proba spectral regions in which turbidity can be retrieved in inland waterbodies using field spectroscopy. Int. J. Remote Sens..

[82] Mandanici E.B.G. (2016). Preliminary comparison of sentinel-2 and landsat 8 imagery for a combined use. Remote Sens..

[83] Gower J.F.R. (1980). Observations of in situ fluorescence of chlorophyll-a in Saanich Inlet. Bound.-Layer Meteorol..

[84] Alikas K.K.K., Reinart A. (2010). Detecting cyanobacterial blooms in large North European lakes using the Maximum Chlorophyll Index. Oceanologia.

[85] Kravitz M.M.J., Stewart B., Derek G. (2020). Application of sentinel 3 OLCI for chl-a retrieval over small inland water targets: Successes and challenges. Remote Sens. Environ..

[86] UNEP GEMStat. https://gemstat.bafg.de/applications/public.html?publicuser=PublicUser#gemstat/Stations.

[87] Oyama Y. (2014). Distinguishing surface cyanobacterial blooms and aquatic macrophytes using Landsat/TM and ETM+ shortwave infrared bands. Remote Sens. Environ..

[88] Hernández-Escobedo Q., Manzano-Agugliaro F., Zapata-Sierra A. (2010). The wind power of Mexico. Renew. Sustain. Energy Rev..

[89] Anatoliy F., Tereshchenko I., Cesar M., Avalos-Cueva D., Pantoja-González D., Rashed M.N. (2016). Climatic Change in a Large Shallow Tropical Lake Chapala, Mexico. Lake Sciences and Climate Change.

[90] Moses W.J., Ogashawara S.S., Montes M.J., De Keukelaere L., Knaeps E., Mishra O.I. (2017). Biooptical Modeling and Remote Sensing of Inland Waters: Atmospheric Correction for Inland Waters.

[91] Mobley C.D. (1999). Estimation of the remote-sensing reflectance from above-surface measurements. Appl. Opt..

